# Plain Words on Registration.—VI
*For previous articles see March 31, p. 521, April 7, p. 15, April 14, p. 35, April 21, p. 53, and May 12, p. 115.


**Published:** 1917-05-19

**Authors:** 


### THE MATRONS' AND SISTERS' DEPARTMENT.

### PLAIN WORDS ON REGISTRATION.—VI.*

### The Value of a State Register.

A COMMON BOND.

IN recent issues we have endeavoured, possibly in somewhat ruthless manner, to demonstrate to those who have allowed themselves to be mesmerised that State Registration, standing alone, is powerless to effect reform, especially in such a profession as that of nursing, where the law fails to interfere with the employment of the unqualified. But the earmarking of appointments for registered nurses only will eventually follow the passing of the Act. Also, in process of time the prohibition of fees for unskilled, as if it were skilled, labour. The true value of such a Register has become lost in party conflict, and a large number of people, vitally concerned, have suffered their vision to be obscured, so that it has become difficult, if not impossible, for them to distinguish that which is true in a confused tangle of fable and imagination.

Nevertheless, a State Register possesses intrinsic value, always provided that it is not regarded as the final goal, but as a means to an end. The end, in a word, is betterment. That which helps the wayfaring nurse along the path of progress is always to be desired. It needs no demonstration that a high educational ideal leads to a higher plane, and this can best be realised in the nursing profession when its members are united by a binding link. Under any Act rules will be made regulating the course of training which a nurse will pursue before she is eligible for enrolment on a State Register. It will be for the nurses themselves, through their representatives on the Council under the Act, to keep the standard high.

THE IMPERIAL IDEAL.

At present, owing to lack of registration, there is no cohesion. Those engaged in hospitals tend to form cliques, while those who are private nurses and unattached become more and more isolated. Registration will supply the bond, and, as our remarks in previous articles on this subject will suggest, the same result may be achieved by any comprehensive system of registration, always provided that it is universally supported. Let us suppose, for example, that owing to some unforeseen contingency legislation were indefinitely postponed, it would be foolish and retrogressive to suspend registration with the Royal British College of Nursing, which has in contemplation a scheme much wider than State Registration. In the Bill promoted by the College it will be noted that the qualifications for registration are those laid down by the College of Nursing. It will be for the College to see that its educational ideals for the profession are achieved. The College ideal includes the most distant parts of the Empire, and herein lies its strength. State Registration will serve to consolidate the profession at home, and the College will bring it into union with India, Canada, New Zealand, Australia. All this has a sentimental value, and sentiment is by no means a worthless thing. But in addition there is substantial material betterment involved, and this ought to appeal especially to the younger generation. What a priceless comfort to an emigrant nurse from the Homeland as from the Colonies to find herself at her journey's end among her sisters in a club of which she had previously been a member, and with whose aims and privilege she was already in full sympathy! If the entire profession were organised and consolidated, this ideal would be nearer realisation. And it is because the foundation-stone of the State is the strongest and the most firmly set in our national and imperial economy that it is desirable, if possible, to establish a Nurses' Register under its authority.

THE OUTLOOK.

Let it never be forgotten, in considering either a State Register or an Imperial College, that material betterment is the ultimate goal. Better remuneration, better hours of labour, longer holidays, travelling privileges, holiday homes, a strong benevolent and pension fund—these are the tangible things that appeal to hardworking women, and they are each and every one attainable. The working life of a nurse is exceptionally brief, exceptionally onerous. Roundly, she enters on her career at twenty-two, she begins to disappear at forty-two. The improving of her outlook is therefore a work of urgent necessity. Perhaps the most powerful help that Registration can indirectly bring to the cause is the enlisting and the strengthening of public opinion in the nurse's favour. The fact is not sufficiently appreciated that a nurse's life is one of constant sacrifice for the public benefit, and therefore when the public are asked to provide a fund for the benefit of British nurses it must be kept prominently in view that this is not the bestowal of charity, but the partial discharge of a heavy debt. The man in the street owes to the sister in the wards more than he wots of, a fact which must be sounded with trumpet-note lest it be forgotten. The man in the street is not unreasonable when he is well informed. The consolidation of the nursing profession must be instrumental in concentrating nursing public opinion, a force now largely diffused and wasted.

We trust that our efforts have been instrumental in separating the corn from the chaff. As we stated in our opening remarks, we shall rejoice when State Registration is an accomplished fact, mainly because it will enable us to proceed with other necessary reforms. We cannot too persistently iterate that these reforms are urgent, and that there is serious danger of their being overlooked by regarding Registration as a gateway to El Dorado.

* For previous articles see March 31, p. 521, April 7, p. 15, April 14, p. 35, April 21, p. 53, and May 12, p. 115.

